# INCOME POOLING IN MIDLIFE: A COMPARISON OF REMARRIED AND COHABITING RELATIONSHIPS

**DOI:** 10.1093/geroni/igae098.1189

**Published:** 2024-12-31

**Authors:** Matthew Wright, Tatum Schwartz, Susan Brown, Wendy Manning

**Affiliations:** Appalachian State University, Boone, North Carolina, United States; University of California Los Angeles, Los Angeles, California, United States; Bowling Green State University, Bowling Green, Ohio, United States; Bowling Green State University, Bowling Green, Ohio, United States

## Abstract

The share of midlife cohabitors has risen in recent decades, though little is known about their income pooling practices. Cohabitation could be attractive because partners may be able to preserve their economic autonomy and maintain assets for the next generation. Conversely, cohabitation may operate as an alternative to marriage, allowing couples to combine their resources to achieve economies of scale without the legal obligations of marriage. We used the nationally representative Families and Relationships Survey to compare income pooling among cohabiting and remarried U.S. adults aged 50-65 (N = 888). Aligning with the hypothesis that cohabitation and remarriage are distinct in middle age, the odds of income pooling were lower for cohabitors than remarrieds. However, the gap between cohabitors and remarrieds narrowed by later ages. This study provides insight into the economic organization of midlife cohabiting relationships, which may have implications for individual well-being and relationship decision-making among middle-aged couples.

